# The Effects of Cold Water Immersion on the Recovery of Drop Jump Performance and Mechanics: A Pilot Study in Under-20 Soccer Players

**DOI:** 10.3389/fspor.2020.00017

**Published:** 2020-03-31

**Authors:** Adam Kositsky, Janne Avela

**Affiliations:** Biology of Physical Activity, Neuromuscular Research Center, Faculty of Sport and Health Sciences, University of Jyväskylä, Jyväskylä, Finland

**Keywords:** biomechanics, exercise, fatigue, cryotherapy, hydrotherapy

## Abstract

Cold water immersion (CWI) is a popular method used for enhancing recovery from exercise. However, the efficacy of this approach is inconclusive and studies investigating variables contributing to overall performance are scarce. Additionally, few studies have investigated the recovery of stretch-shortening cycle (SSC) performance after a fatiguing SSC task. The SSC occurs naturally in human locomotion and induces a recovery pattern different from isolated muscle contractions (e.g., pure eccentric exercise). Therefore, the main aim of this study was to investigate the effects of a single CWI on jumping performance and mechanics after exhaustive SSC exercise. On a sledge apparatus, 10 male under-20 soccer players (age 18–20 years) performed five sets of 20 maximal drop jumps (DJ) followed by continuous submaximal rebounding. Subjects were equally randomized into a passive recovery control (CON) or CWI group (10 ± 0.5°C for 20 min). Prior to, upon completion of, and at 24 and 48 h follow-ups, subjects performed maximal DJs recorded with a high-speed video camera. Blood samples were taken and subjective muscle soreness was measured. Rebound jump height was impaired immediately after exercise, although significant only for CWI (CON: −12.4 cm, *p* = 0.083; CWI: −9.9 cm, *p* = 0.009). The CWI group demonstrated significant recovery of jump height at 24 h (+6.3 cm, *p* = 0.031) and 48 h (+8.9 cm, *p* = 0.002) compared to post-exercise. Ankle joint stiffness was decreased for CWI (−2.1 to −2.5 Nm/°, *p* = 0.005–0.041). Creatine kinase activity was similarly increased for both groups at 24 and 48 h, while there was also no group effect in muscle soreness (*p* ≥ 0.056). This pilot study demonstrates the potential for CWI to slightly enhance the recovery of DJ performance. However, this occurred in parallel with reduced ankle joint stiffness, signifying that jumps were performed with less efficiency, which would not be favorable for repeated SSC actions. While this should be confirmed with a larger sample size, this highlights the potential for CWI to be detrimental to the mechanical properties of the ankle joint. Therefore, future recovery intervention studies should concomitantly investigate variables contributing to performance, rather than just overall performance itself.

## Introduction

Athletes, particularly those at elite levels, require peak performance levels on a daily basis. When subsequent training sessions, matches, or competitions occur before recovery is complete, short-term overreaching and long-term overtraining may occur, resulting in non-optimal neuromuscular function and diminished performance (Meeusen et al., 2013). Consequently, numerous recovery methods are implemented with the aim of enhancing restoration of the neuromuscular system. One of the most popular approaches is cold water immersion (CWI), a form of cryotherapy thought to be effective by reducing the metabolic rate and inflammatory response of damaged musculoskeletal tissues (Wilcock et al., 2006).

However, although there is an abundance of literature, the evidence is inconclusive on the efficacy of CWI for recovery of performance. Ascertaining the effectiveness of CWI remains difficult owing to inconsistency and little standardization across studies, complicating comparison between previous literature (Anderson et al., 2018). In addition to a wide variation in the temperature, duration, and depth (e.g., whole- or lower-body) used in CWI protocols, subject demographics (e.g., training status, gender, etc.) and choices of fatiguing-exercise also need to be considered (Higgins et al., 2017). Further, few studies have investigated the effects of a single CWI after stretch-shortening cycle (SSC) exercise. The SSC sequence consists of an active muscle-tendon unit lengthening prior to shortening, resulting in enhanced performance during the shortening phase (Komi, 2000; Nicol et al., 2006). The SSC occurs in typical locomotive tasks (e.g., walking, running, jumping, etc.) and thus repeated SSC exercise is a way to study natural neuromuscular fatigue (Komi, 2000; Nicol et al., 2006).

Repetitive SSC exercise decreases jumping performance and increases metabolic activity (Nicol et al., 2006). However, fatigue-related changes after SSC exercise are dependent on the task tested (Nicol et al., 2006). Unfortunately, most studies that have used CWI after repeated SSC exercise have generally investigated recovery of performance during a task different to that used in the fatiguing protocol (Howatson et al., 2009; Jakeman et al., 2009; White et al., 2014; Leeder et al., 2015; Vieira et al., 2016; Anderson et al., 2018; Ahokas et al., 2019). To our best knowledge, only Skurvydas et al. (2006) investigated the recovery of drop jump (DJ) performance after using repeated DJs as an exercise task, observing that CWI can improve the recovery of DJ rebound height compared to passive recovery. Nonetheless, multiple immersions were used (Skurvydas et al., 2006), which may be inadvisable as a long-term strategy (Roberts et al., 2015). Thus, it is unclear if a single CWI would also be beneficial for the recovery of DJ rebound height.

SSC fatigue also produces changes in lower-limb kinematics and kinetics (Nicol et al., 1991; Mizrahi et al., 2000; Kuitunen et al., 2002; Chappell et al., 2005; Weinhandl et al., 2011). These modifications to movement strategies are thought to be a risk for musculoskeletal injury (Mizrahi et al., 2000; Chappell et al., 2005); however, little is known about how CWI affects joint biomechanics. Acutely after CWI applied in a non-fatigued state, there have been minimal changes to lower-limb kinematics and kinetics (Wang et al., 2010; Fukuchi et al., 2015). To our best knowledge, no studies have yet investigated the effects of CWI on ankle joint (AJ) biomechanics after fatigue. Additionally, less is known about how CWI may affect the efficiency of movement. Joint stiffness is an important parameter for impact loading and elastic energy use (Hoffrén et al., 2007; Yoon et al., 2007) that has been reported to decrease with SSC fatigue (Kuitunen et al., 2002). It may then be that CWI enhances the recovery of joint stiffness, allowing for improved SSC performance.

In this pilot experiment, we investigated the effect of CWI on both performance and perceived fatigability (Enoka and Duchateau, 2016) after exhaustive SSC exercise consisting of repeated maximal DJs and submaximal rebounding. Changes in maximal DJ rebound jump height, the associated movement mechanics, and creatine kinase (CK) were considered performance fatigability and measures of muscle soreness were taken for perceived fatigability. We hypothesized that the exercise task would hamper rebound jumping performance and technique and elevate CK activity and muscle soreness. It was further hypothesized that CWI would enable a faster recovery of both performance and perceived fatigability.

## Methods

### Subjects

Ten male soccer players (age 18–20 years) from the under-20 team of a club that had recently been promoted to the Finnish premier division (Veikkausliiga) participated. Subjects were requested to not perform any strenuous activity starting from 48 h prior to the first day of testing and lasting until the completion of the testing. Subjects were randomized into either a controlled passive recovery group (CON; *n* = 5; mean age = 18.4 ± 0.5 years; mean height = 182.5 ± 7.2 cm; mean body mass = 74.9 ± 5.4 kg) or CWI (*n* = 5; mean age = 19.4 ± 0.9 years; mean height = 182.0 ± 5.9 cm; mean body mass = 76.9 ± 8.1 kg). The University of Jyväskylä Ethical Committee, in accordance with the Declaration of Helsinki, approved the study. Written consent was provided prior to the investigation commencing.

### Design

**Figure 1:**
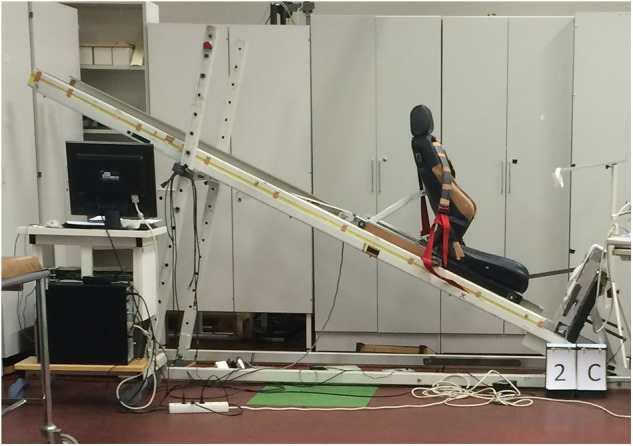
Sledge apparatus used for drop jumps.

The experiment was designed as a pre- and post-SSC fatigue follow-up study on a sledge apparatus (Dousset et al., 2007). The sledge was inclined at 24.9° with respect to the horizontal plane (Figure 1). SSC fatigue was chosen because it forms the basis for natural human locomotion (Komi, 2000; Nicol et al., 2006) and is thus relevant to most elite and recreational athletes. Additionally, exhaustive SSC exercise typically induces a bimodal recovery model that differs from other modes of fatigue. Briefly, the bimodal pattern of recovery from fatiguing SSC exercise shows an acute recovery (2 h) after an immediate observation of fatigue at POST before further deterioration at 24/48 h (Nicol et al., 2006). The initial impairment is due to metabolic fatigue while the secondary decline is due to muscle damage (Nicol et al., 2006).

Subjects visited the lab four times, the first being a familiarization session at least 1 week prior to the fatiguing jumps. Baseline measurements (PRE) were compared with those taken immediately after (POST) exhaustive SSC exercise and at 24 and 48 h. All subjects completed identical protocols, with the only difference being the recovery intervention. To investigate the efficacy of the recovery method, subjects performed maximal DJs on a sledge ergometer, reported subjective values of muscle soreness, and had fingertip blood samples taken. Data collection occurred during their off-season in order to limit the influence of in-season fatigue. However, due to the demands of testing and a limited time window, we were not able to have a repeated-measures cross-over design with subjects performing the protocol with both recovery interventions.

### Methods and Procedures

The optimal dropping height on the sledge apparatus was determined during the familiarization visit. Subjects were dropped from increasing 10 cm intervals until rebound height ceased to increase. The dropping height that produced the largest rebound height was determined to be the optimal dropping height.

The fatigue protocol occurred during the second visit, with measurements at PRE and POST. After a 10 min standardized cycle ergometer warm-up (Regueme et al., 2005), subjects performed 100 successive maximal bilateral DJs from the optimal dropping height (Kuitunen et al., 2002, 2004; Nicol et al., 2003; Dousset et al., 2007) divided into five sets of 20 with 2 min rest between sets (Miyama and Nosaka, 2004). This protocol has been shown to induce muscle damage (Kuitunen et al., 2002; Miyama and Nosaka, 2004; Dousset et al., 2007; Piitulainen et al., 2008) and has been used in other CWI recovery studies (Howatson et al., 2009; Vieira et al., 2016). Five seconds were given between each successive maximal DJ (signaled via an audible metronome). Upon completing the final set, to induce muscle fatigue (i.e., metabolic loading) subjects immediately began continuous submaximal rebounding to 70% of their maximal rebound height (Kuitunen et al., 2002, 2004; Nicol et al., 2003; Dousset et al., 2007) and continued until they could not maintain at least 50% of maximal rebound height or volitional fatigue. Fatigue of the triceps surae was maximized, and hip and knee extensor fatigue minimized, by inclining the seat to 120°, allowing knees to flex freely during the rebound airborne phase, requesting maximal knee angle during ground contact be limited to ~90°, and keeping heels off the force platform (Regueme et al., 2005; Dousset et al., 2007). For all jumps, subjects were instructed to explosively rebound by jumping as high and as fast as they could.

After finishing POST measurements, subjects were randomized into CON or CWI. The CWI consisted of 20 min with the lower legs immersed in a bucket filled with cold water maintained at 10 ± 0.5°C by crushed ice. This temperature and duration has been shown to decrease triceps surae intramuscular temperature by ~5°C (Myrer et al., 1998). To limit the effects of the immersion to the triceps surae and AJ, the immersion depth was set to the level of the popliteal crease. During CWI, neoprene thermal sleeves (Meister, Minneapolis) covered the distal foot to reduce discomfort and pain sensation (Misasi et al., 1995). The CON group sat with their lower legs in an empty bucket for 20 min to represent a seated, passive recovery. Subjects returned to the lab 24 and 48 h after completion of the fatiguing task to perform follow-up measurements.

### Measurements

Maximal DJs on the sledge apparatus were conducted as a performance test. Upper body involvement was limited by folding arms across the chest during jumps. Jump height was calculated as the change in sledge position from standing to peak rebound. Ground reaction force (F_z_) was sampled at 1,000 Hz (CED 1401, Cambridge Electronics Design, Cambridge, UK) with a rigid force plate situated perpendicular to the slope of the sledge apparatus and analyzed using Spike2 software (version 6.17, Cambridge Electronics Design, Cambridge, UK). The highest F_z_ measured immediately after impact was recorded as the peak impact force.

Reflective markers were placed over the greater trochanter, lateral femoral condyle, lateral malleolus, heel, and base of the fifth metatarsal of the right lower limb. A high-speed camera (Sony NXCAM, HXR-NX5E, Japan) recorded at 200 frames per second from the subject's right side and an LED light, which signaled when force was above baseline, was used for synchronization purposes. Two-dimensional (sagittal plane) motion analysis video clips were converted to .AVI format using Kinovea (version 0.8.15, Kinovea Org., France). Markers were then digitized and processed using Vicon Motus (version 10.0, Vicon Motion Systems, Oxford, UK). Reflective markers were placed at a known distance alongside the measuring tape on the sledge apparatus for calibration purposes. The jumps were divided into two phases: the braking (eccentric) phase was defined as the segment between the frame where the toes first contacted the force plate (i.e., the LED light brightened) and the lowest point (i.e., sledge seat no longer displaced downwards), and the push-off (concentric) phase was defined as the segment between the lowest point and the frame where the toes left contact with the force plate. Knee joint (KJ) angle was calculated between the greater trochanter and lateral malleolus with the lateral femoral condyle as the pivot marker. AJ angle was a segmental angle between lateral femoral condyle and base of the fifth metatarsal, using both the lateral malleolus and heel markers as centers of rotation. A 20 Hz low-pass Butterworth filter was applied to the raw coordinates and the average of 2–3 trials were used. For the AJ, 90° represented neutral angle with higher values indicating plantarflexion, and 180° represented full KJ extension.

The AJ moment was estimated (Kawakami et al., 2002; Hoffrén et al., 2007; Kubo et al., 2007):

$$\begin{array}{l} \text{Moment}={\text{F}}_{\text{z}}{\;}^{*}{\text{L}}_{\text{Foot}}{\;}^{*}\text{cos}(\theta_{\text{AJ}}-90) \end{array}$$

where F_z_ is the ground reaction force (as explained above), L_Foot_ is the estimated length between the center of the ankle joint and the ball of the foot, and θ_AJ_ is the ankle joint angle at the lowest point. The change in AJ moment divided by the change in AJ angle at the end of the braking phase calculates AJ stiffness (Kuitunen et al., 2002).

Blood samples were drawn from separate digits after cleansing the fingertips with sanitizing alcohol. Lacate was analyzed instantly and automatically (Lactate Scout, SensLab GmbH, Germany), while blood samples were stored in tubes and refrigerated for later CK activity analysis (Konelab 20XTi, Thermo Fisher Scientific Oy, Finland). Measurements of triceps surae muscle soreness were taken from maximal DJs. After jumping, the subjects were asked to draw a straight line on a visual analog scale from 0 to 10 cm, where 0 represented no pain and 10 represented severe pain.

### Statistical Analysis

A Shapiro-Wilk test was performed to assess normal distribution. Because of the low sample size per group and instances of non-normally distributed data, mixed methods ANOVA was not used (Oberfeld and Franke, 2013). For normal data, dependent *t*-tests were used to assess within-group differences and independent *t*-tests were performed to assess between-group differences. If the data were not normal, they were replaced by Wilcoxon Signed Rank and Mann-Whitney *U*-tests, respectively. If two pieces of data from the same variable had opposing normality (e.g., normal at PRE and non-normal at POST), the non-normal tests were used. These variables were CK activity, muscle soreness, peak impact force, and KJ and AJ angle at initial contact. Hedges' *g* effect sizes with a correction for a small sample bias were calculated for between-group effects (Durlak, 2009) and interpreted as ≥ 0.20 = small, ≥ 0.50 = medium, and ≥ 0.80 = large. Unless otherwise noted, data are presented as means ± standard deviations (SD). Statistical significance was set at *p* < 0.05 and confidence intervals at 95%. As this study was exploratory in nature, corrections for multiple comparisons were not performed (Bender and Lange, 2001). A CWI subject was excluded for AJ stiffness calculations as the force during the whole braking phase at POST could not be determined due to errors in the signal.

## Results

**Figure 2:**
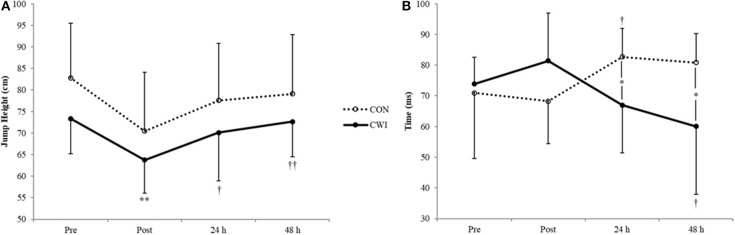
Mean values (± SD) in **(A)** rebound height and **(B)** time to peak impact force during maximal DJ test. *(*p* < 0.05) between groups, **(*p* < 0.01) from PRE; ^†^(*p* < 0.05), ^†^^†^(*p* < 0.01) from POST.

Hedges' *g* effect sizes are summarized in Table 1. There was no statistically significant difference between groups in the number of jumps performed (CON: 188 ± 24; CWI: 377 ± 234; *p* = 0.145). Both groups had large decreases in maximal rebound height at POST, but only CWI was significantly different (CON: *p* = 0.083; CWI: *p* = 0.009) (Figure 2A). The large SD for CON was the result of one subject jumping 0.4 cm higher POST. In terms of change from POST, CWI showed significant signs of recovery at both 24 h (*p* = 0.031) and 48 h (*p* = 0.002) while CON did not (24 h: *p* = 0.156; 48 h: *p* = 0.124). The non-significant change for CON was mostly likely due to no significant difference being found even at POST, as no group differences were found at any timepoint (*p* ≥ 0.212, *g* = 0.03–0.51). The time to peak impact force did not significantly change at POST (Figure 2B). However, relative to POST there were significant differences at 24 h for CON (*p* = 0.028) and 48 h for CWI (*p* = 0.036). The group differences between these changes from POST were *p* = 0.033 (24 h; *g* = 1.31) and *p* = 0.012 (48 h; *g* = 1.65). The peak impact force itself was significantly increased at POST for CWI (130.5 ± 115.6 N; *p* = 0.043), with no group difference at any timepoint (*p* ≥ 0.548; *g* = 0.05–0.45).

**Table 1:** Hedges' *g* between-group effect sizes across different timepoints.

	**Pre-Post**	**Pre-24 h**	**Pre-48 h**	**Post-24 h**	**Post-48 h**	**24–48 h**
Jump height	0.22^S^	0.26^S^	0.51^M^	0.09	0.03	0.28^S^
Peak impact force	0.34^S^	0.05	0.27^S^	0.16	0.18	0.45^S^
Time to peak impact force	0.64^M^	0.94^L^	1.00^L^	1.31^L^	1.65^L^	0.25^S^
KJ angle initial contact	0.38^S^	0.92^L^	0.68^M^	0.37^S^	0.30^S^	0.13
AJ angle initial contact	0.01	0.22^S^	0.29^S^	0.33^S^	0.39^S^	0.10
KJ angle takeoff	0.80^L^	3.12^L^	0.55^M^	0.21^S^	0.19	0.39^S^
AJ angle takeoff	0.22^S^	0.57^M^	0.14	0.39^S^	0.19	0.73^M^
AJ stiffness	0.38^S^	1.19^L^	1.07^L^	0.60^M^	0.44^S^	0.27^S^
Muscle soreness	0.05	0.34^S^	0.10	0.78^M^	0.18	0.77^M^
Creatine kinase	0.38^S^	0.62^M^	0.09	0.57^M^	0.16	0.55^M^

AJ, angle joint; KJ, knee joint; S, small; M, medium; L, large.

**Figure 3:**
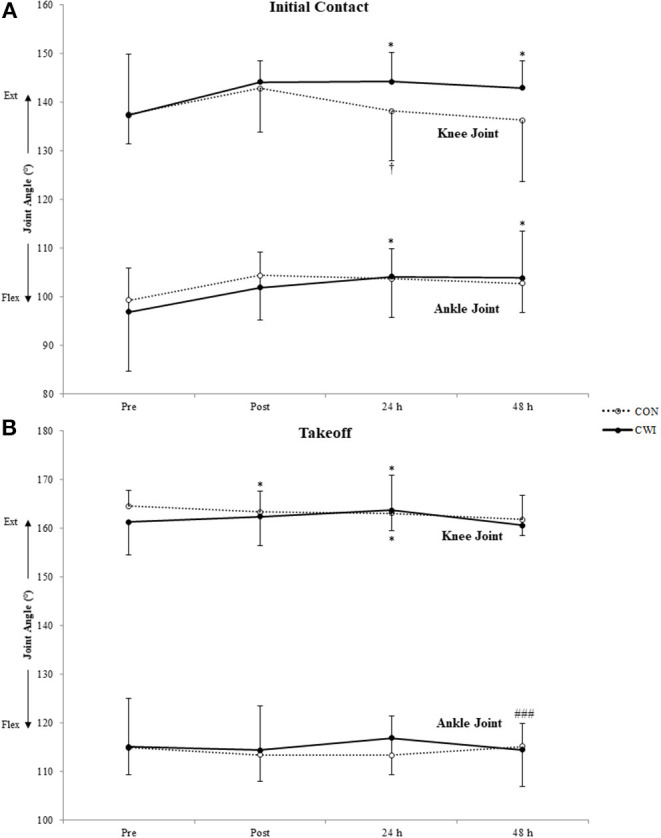
Mean values (± SD) of knee and angle joint angle. Higher values represent joint extension. **(A)** Initial contact. **(B)** Takeoff. *(*p* < 0.05) from PRE, ^†^(*p* < 0.05) from POST, ^*###*^(*p* < 0.001) from 24H.

**Figure 4:**
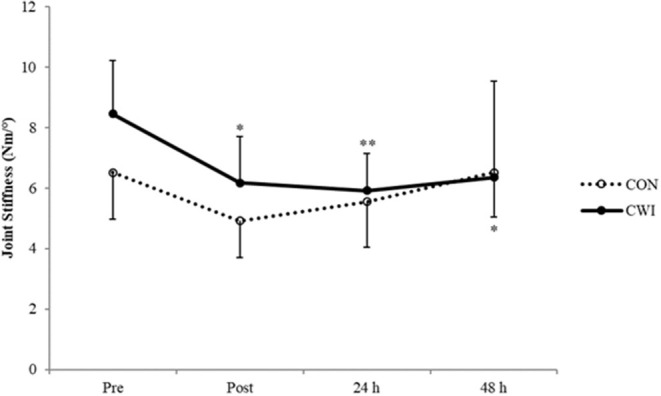
Mean values (± SD) of ankle joint stiffness. *(*p* < 0.05), **(*p* < 0.01) from PRE.

The AJ angle was more plantarflexed at initial contact for CWI at 24 and 48 h (both *p* = 0.043) (Figure 3A), while at 48 h it was more plantarflexed at takeoff for CON compared to 24 h (*p* < 0.001) (Figure 3B). The KJ angle was increased at initial contact for CWI at 24 and 48 h (both *p* = 0.043) and decreased at 24 h relative to POST for CON (*p* = 0.043) (Figure 3A). No significant changes were evident in KJ (CON: *p* ≥ 0.130; CWI: *p* ≥ 0.127) or AJ (CON: *p* ≥ 0.581; CWI: *p* ≥ 0.064) angle at the lowest position. The KJ angle at takeoff was significantly decreased for CON at POST (*p* = 0.011) and 24 h (*p* = 0.040) while it was significantly increased for CWI at 24 h (*p* = 0.007) (Figure 3B). AJ stiffness was significantly decreased at follow-up timepoints for CWI (*p* = 0.005–0.041) (Figure 4). Although no significant differences were found for CON (*p* ≥ 0.101), at POST all but one of the subjects displayed decreases in AJ stiffness. No between-group differences were found for AJ or KJ angle at any phase of the DJ (*p* ≥ 0.286). While there was no significant difference in AJ stiffness between groups (*p* ≥ 0.176), a large effect in the change from baseline was evident at 24 h (*g* = 1.19) and 48 h (*g* = 1.07).

**Figure 5:**
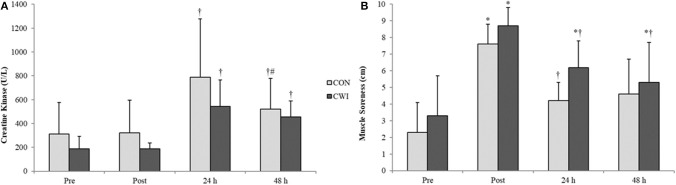
Mean values (± SD) of **(A)** creatine kinase activity and **(B)** muscle soreness before and after exercise. *(*p* < 0.05) from PRE, ^†^(*p* < 0.05) from POST, ^#^(*p* < 0.05) from 24H.

Lactate was significantly increased post-exercise for both groups (CON: mean difference 10.6 ± 2.2 mmol/L, *p* < 0.001; CWI: mean difference 9.0 ± 3.2 mmol/L, *p* = 0.003). Relative to POST, CK activity was significantly increased for both groups at 24 and 48 h (all *p* = 0.043) (Figure 5A). There was also a decrease in CK activity between 24 and 48 h for CON only (*p* = 0.043). Muscle soreness was significantly increased for CWI at all follow-up timepoints (*p* = 0.042–0.043), but only at POST for CON (*p* = 0.043) (Figure 5B). There were no between-group differences for lactate (*p* ≥ 0.461; *g* = 0.46), CK activity (*p* ≥ 0.301; *g* = 0.09–0.62), or muscle soreness (*p* ≥ 0.056; *g* = 0.05–0.78).

## Discussion

The purpose of the study was to investigate changes in DJ performance and mechanics after exhaustive SSC exercise on a sledge apparatus and to follow the recovery patterns of two groups, CON and CWI at 10°C for 20 min. The results show an immediate effect of exercise on DJ performance, with large decreases in rebound height at POST. Secondly, it appears that CWI may slightly enhance the recovery of DJ performance, although AJ stiffness remained significantly decreased. Evidence of muscle damage progressed throughout the experiment, and there were few evident statistical differences between intervention groups for all parameters. Only time to peak impact force at 24 and 48 h differed between groups, with CWI reaching their impact peak in a shorter duration.

Subjects performed less jumps compared to other maximally exhaustive SSC exercise investigations (Kuitunen et al., 2004; Piitulainen et al., 2008). In the present study, subjects ceased jumping upon volitional fatigue or if they could not maintain a rebound height of 50% of maximal, whereas subjects previously (Kuitunen et al., 2002, 2004; Nicol et al., 2003; Dousset et al., 2007; Piitulainen et al., 2008) continued rebounding until they “were completely exhausted so that they were unable to jump off the force plate of the sledge” (Kuitunen et al., 2004). While such fully exhaustive protocols are recommended to reduce inter-subject variability in fatigue (Nicol et al., 2006), they rarely occur in sporting environments and thus we deemed it not practically applicable to use such a protocol. On the other hand, the number of jumps and the decrements in rebound jump height at POST were comparable with other studies that have used a submaximal exhaustive SSC protocol (Regueme et al., 2007; Morio et al., 2011, 2012). Further, although the number of jumps between groups was not significantly different, there were two subjects in the CWI group that performed a considerable number of jumps (510 and 726) compared to the other eight subjects across both groups (range: 164–241). While this could have led to an unequal group distribution of “fast-exhausted” and “slow-exhausted” jumpers (Kuitunen et al., 2004), lactate increased similarly for both groups at POST, demonstrating metabolic loading from the exercise was comparable at group level. Using POST as a baseline for recovery, increases in rebound jump height were found at 24 and 48 h for CWI only, although that is likely due to CON not being significantly different at POST as a result of one subject jumping 0.4 cm higher. However, this suggests CWI may potentially have a beneficial effect compared to CON on jump height, comparable with previous observations after multiple CWIs (Skurvydas et al., 2006). After fatiguing high-intensity sprints, a single CWI at 10°C for 10 min enhanced the recovery of DJ performance at 24 and 48 h (White et al., 2014). Nonetheless, at POST the CON group decreased jump height by ~3 cm more than the aforementioned CWI group (Figure 6 of White et al., 2014). In that study, the relative change from POST (i.e., recovery from fatigue) followed a similar pattern to the CWI group, which corroborates with our results and supports only a medium effect for CWI on rebound jump height.

After repetitive DJs, greater ankle plantarflexion occurs at initial contact in the presence of greater knee extension (Weinhandl et al., 2011). In the present study, both groups demonstrated a trend in this direction at POST, although values were not significantly different from PRE. However, the CWI group landed with more extended joints at 24 and 48 h, leading to a reduction in AJ stiffness at these timepoints, where the between-group effect was large. Previously, AJ stiffness remained reduced at 48 h (considered as CON subjects as no recovery intervention was used) (Kuitunen et al., 2002). Contrarily, in the present study AJ stiffness remained decreased at 24 and 48 h for the CWI group only. One explanation that the authors (Kuitunen et al., 2002) offered was that joint stiffness decrements could be related to muscle damage. Indeed, muscle soreness was elevated only for CWI. Furthermore, unlike our study, Kuitunen et al. (2002) additionally focused on fatigue at the KJ. It is possible that coupling effects with quadriceps fatigue keeps AJ stiffness reduced. It is difficult to make concrete conclusions on this as we did not measure KJ stiffness in the present study. On the other hand, CWI to knee level has previously demonstrated a significant reduction in AJ impact absorption during landing (Wang et al., 2010). This is comparable with the present study as decreased AJ stiffness was clear only in the CWI group. Regardless of the origin of the reduction in AJ stiffness, this has important implications since reduced AJ stiffness results in less efficient SSC performance as elastic energy cannot be sufficiently stored and released (Yoon et al., 2007). The influence of joint stiffness is less important in single movements (Kubo et al., 2007), thus the decreased efficiency may be negligible for a few trials. However, if CWI after fatiguing SSC actions does generally lead to decreased SSC efficiency, this would then be extremely relevant to athletes.

Exhaustive SSC exercise on a sledge apparatus has previously been shown to significantly increase muscle soreness that remains elevated for at least 48 h (Nicol et al., 2003; Piitulainen et al., 2008). Studies that have compared CON and CWI after SSC exercise have found that active muscle soreness is increased for both groups at both 24 and 48 h after single (Leeder et al., 2015; Vieira et al., 2016; Anderson et al., 2018; Ahokas et al., 2019) and multiple (Howatson et al., 2009) immersions. In the present study, both groups had increases in CK activity at 24 and 48 h, with a moderate effect for greater CK activity in the CON group. However, the absolute value of CK activity may not accurately reflect the magnitude of muscle damage (Fridén and Lieber, 2001). Indeed, at 24 and 48 h only CWI had higher muscle soreness compared to baseline. It is unclear why our results differ from previous studies, since the subjects in Leeder et al. (2015) were also well-trained team sport athletes. Although this did not seem to aid performance for the CON group, it may be that the higher muscle soreness during DJ in the CWI group led to an alteration in DJ mechanics, in turn reducing the efficiency of the movement.

## Limitations

Caution does need to be taken when applying results from this pilot experiment. Firstly, the sample size was relatively low, especially pertaining to individual groups, which limited the statistical analyses that could be performed. Both factors may have led to Type II errors. Additionally, due to a short off-season time window (~1 month) and large demands of testing (four visits per subject), it was not feasible to have subjects repeat the protocol in a cross-over design. Secondly, data on long-term effects of a single CWI are scarce, and longitudinally applying results from acute investigations may not be correct. Thirdly, a rationale could be made that because the study was mainly interested in the plantarflexors and the AJ, knee movement should have been completely restricted. However, because the gastrocnemii muscles cross the KJ, it was necessary to allow knee flexion during DJs. As such, we took extra precaution to minimize the amount of involvement and fatigue of the other muscles spanning the KJ (Regueme et al., 2005; Dousset et al., 2007). Additionally, the immersion was kept below KJ level in order to establish effects purely due to cooling of the lower leg and ankle and to eliminate possible confounders, such as the effects of cooling the KJ and patellar tendon (Alegre et al., 2016). Finally, we only measured two-dimensional kinematics from one leg in a bilateral task. However, the movement plane of the sledge is strictly sagittal, and the lab set-up constrained the use of three-dimensional motion capture. Further, although asymmetrical motor responses have been reported after single limb CWI (Delahunty et al., 2019), it is unknown if this is long-lasting and a bilateral immersion likely negated these effects.

## Conclusions

The main finding from this pilot study was that the slightly enhanced recovery of maximal DJ performance after post-exercise CWI may be at the expense of efficiency, as decreased AJ stiffness was also found. This would be unfavorable during repeated SSC actions. Although this should be confirmed with a larger sample size, it appears that the possible benefits of CWI are most likely attributable to factors other than the mechanical properties of the AJ, which in fact may be quite detrimental in nature. These results highlight and emphasize the need for future recovery intervention studies to investigate not only the global output after an intervention, but also the neuromuscular mechanics contributing to that performance in order to determine exactly which underlying mechanism(s) may lead to the potential benefit(s) of the respective interventions.

## Data Availability Statement

The raw data acquired for this study are available upon reasonable request.

## Ethics Statement

This study involving human participants was reviewed and approved by the University of Jyväskylä Ethical Committee. The subjects provided their written informed consent to participate in this study.

## Author Contributions

AK and JA conceived and designed the research. AK conducted experiments, analyzed and interpreted data, and wrote the initial manuscript draft. JA assisted in data interpretation and manuscript revisal. All authors read and approved the final manuscript.

### Conflict of Interest

The authors declare that the research was conducted in the absence of any commercial or financial relationships that could be construed as a potential conflict of interest.
